# Implementation of Teach-Back method based on KWL strategy: a randomized controlled trial in medical caregiver retraining

**DOI:** 10.3389/fpubh.2026.1785892

**Published:** 2026-06-17

**Authors:** Xintong Zhang, Chunmei Zhao, Yufei Qian, Qinxia Li, Jianhong Ji, Ying Liu

**Affiliations:** 1 Intensive Care Unit, Nantong First People’s Hospital, Southeast University, Nantong, Jiangsu, China; 2 Department of Gastroenterology, Nantong First People’s Hospital, Southeast University, Nantong, Jiangsu, China; 3 Department of Emergency, Nantong First People’s Hospital, Southeast University, Nantong, Jiangsu, China; 4 Department of Infection Management, Nantong First People’s Hospital, Southeast University, Nantong, Jiangsu, China; 5 Department of Nursing, Nantong First People’s Hospital, Southeast University, Nantong, Jiangsu, China

**Keywords:** KWL strategy, loose-leaf teaching material, medical education, nursing education, retraining, Teach-Back teaching method

## Abstract

**Background:**

Effective retraining programs for medical caregivers (i.e., patient care assistants who primarily assist nurses with daily patient care) are critical to ensuring patient safety and care quality. However, traditional lecture-based methods often fail to promote durable knowledge retention and skill application. The aim of this study was to assess the effectiveness of a retraining program integrating the Teach-Back method with the Know–Want to know–Learned (KWL) strategy and loose-leaf teaching materials.

**Methods:**

Medical caregivers undergoing retraining at a hospital between January and June 2025 were randomly assigned to either an experimental group (*n* = 25) or a control group (*n* = 25) using a random number table. The experimental group received instruction through loose-leaf materials incorporating the Teach-Back method guided by the KWL strategy. This approach involved structured guidance to activate prior knowledge (Know), define learning goals (Want to know), and consolidate learned content (Learned), along with real-time feedback delivered through the Teach-Back process. The control group received conventional lecture-based instruction. Outcomes were compared between the groups using theoretical examination scores, practical skill assessment scores, and self-reported competence ratings.

**Results:**

The experimental group demonstrated significantly higher theoretical knowledge and practical skill scores compared with the control group (both *p* < 0.05). Self-evaluated competence indicated notable improvements in theoretical knowledge, operational skills, social competence, and professional attitude within the experimental group.

**Conclusion:**

A retraining program that integrates the Teach-Back method and the KWL strategy with loose-leaf teaching materials can substantially improve learning outcomes, skill application, and self-perceived competence among medical caregivers. This model holds promise for broader application in clinical training for medical caregivers and can also inform continuing education programs in nursing and other healthcare disciplines.

## 1 Overview

Globally, the shortage of nursing personnel remains a key challenge, further compounded by increasing workloads and intensified nursing responsibilities (1). The combined effects of population aging and evolving family structures have worsened the mismatch between available nursing resources and the growing healthcare needs of patients (2). In China, within ongoing healthcare reforms that advocate for “accompany-free” ward management and the provision of high-quality nursing services, imbalances in healthcare resource allocation, workforce distribution, and personnel supply have become increasingly evident (3).

Given the rising demand for diverse care services and the persistent shortfall in clinical personnel, medical caregivers have emerged as a vital supplementary component of the nursing workforce (4). In the Chinese healthcare system, “medical caregivers” are ancillary personnel who provide non-clinical support to patients under the supervision of registered nurses. Their duties include assisting patients with daily activities (e.g., bathing, feeding, mobility) and maintaining ward hygiene. Unlike registered nurses, medical caregivers do not perform clinical assessments, administer medications, or conduct invasive procedures. They serve as a supplementary workforce to alleviate the nursing shortage, particularly in tertiary hospitals. In recent years, Chinese national policy initiatives have emphasized the importance of standardizing caregiver management and training systems and promoting workforce development in this sector to ensure the quality of services provided by medical caregivers, enhance patient care experiences, and address heterogeneous healthcare needs (5, 6).

However, in many countries, including China, the training and management of medical caregivers remain in an exploratory phase (7). Disparities in training standards, inadequacies in assessment frameworks, and limitations in administrative oversight have been documented not only across Chinese regions but also in other healthcare systems. For instance, similar challenges have been reported in the United States (8), Japan (9), and several European countries (10). These persistent issues contribute to inconsistencies in the quality of caregiver services worldwide.

Furthermore, multiple studies have identified low training frequency and limited effectiveness of in-service training and evaluation processes for medical caregivers, often leading to substantial attrition in technical skills over time (11–13). For example, while proficiency in cardiopulmonary resuscitation may be achieved immediately post-training, retention typically declines within 2–6 months (14, 15). In addition, challenges related to the adoption and sustainability of practice changes, such as low motivation to modify established routines and insufficient reinforcement after training, may further contribute to skill decay (16).

Therefore, periodic retraining has been recognized as essential for maintaining and enhancing the overall competency of medical caregivers. Existing literature on caregiver training predominantly addresses curriculum design, development of instructional programs, and the cultivation of core competencies, whereas comparatively less emphasis has been placed on the retraining of theoretical knowledge and practical skills (17–19).

Given the identified problems of skill decay and limited sustainability of practice changes, there is a clear gap in the current retraining approaches for medical caregivers. Most existing programs rely on passive, lecture-based methods that do not ensure comprehension or long-term retention (20). To address this gap, the Teach-Back teaching method, also referred to as the “feedback method,” was introduced. Teach-Back comprises four stages: explanation, evaluation, clarification, and confirmation of understanding (21). It emphasizes active learner engagement and repeated reinforcement, values comprehension-based feedback, and adopts a structured yet accessible instructional approach that is not constrained by the learner’s age or educational background (22, 23). This method has been widely implemented in health education for patients and their families, as well as in professional training for clinical nursing personnel (22–24).

Recently introduced loose-leaf teaching materials present instructional content in a modular format, providing flexibility in instructional design and enabling timely updates (25). These materials serve a dual function, supporting both knowledge dissemination and procedural skill development, thereby addressing the evolving requirements of medical caregiver retraining and professional competency enhancement (25, 26).

The Know–Want to know–Learned (KWL) strategy is a learner-centered, metacognitive framework that promotes structured reflection and supports goal-oriented learning. It guides learners through the completion of a KWL chart, which involves activating existing knowledge (K), identifying learning objectives and needs (W), and summarizing acquired knowledge to promote the development of effective learning strategies (L) (27, 28).

This study was guided by the Information-Motivation-Behavioral Skills (IMB) model, which posits that health-related behavioral change requires information, motivation, and behavioral skills (29). The IMB model has been widely applied in training and education interventions (30, 31). In our retraining program, the IMB model guided intervention design and outcome selection: Information was delivered via online videos and loose-leaf materials; Motivation was enhanced through the KWL strategy; and Behavioral Skills were reinforced via the Teach-Back method. Outcomes included knowledge, operational skills, and perceived competence.

Therefore, when applied during the retraining phase, the Teach-Back method integrated with the KWL strategy and supported by loose-leaf teaching materials can help identify training needs, align instructional content with learner characteristics, enhance training efficiency, and support retention of learning outcomes among medical caregivers. This study aimed to assess the effectiveness of such a retraining program, which integrates the Teach-Back method with the KWL strategy and employs loose-leaf teaching materials, in improving knowledge acquisition, operational proficiency, and perceived competence.

## 2 Participants and methods

### 2.1 Study design

This was a two-arm, parallel-group, randomized controlled trial conducted between January and June 2025. Participants were randomly assigned to either the experimental group (Teach-Back + KWL + loose-leaf materials) or the control group (conventional lecture-based instruction) using a random number table method. The study was registered (ChiCTR2600119467).

### 2.2 Study participants

Using the theoretical knowledge score as the outcome measure, the sample size was calculated using PASS 15 software. Based on pilot study results (*δ* = 1.976, *σ* = 1.862), approximately 20 participants per group were required. Accounting for a 20% attrition rate, a total of 50 medical caregivers who worked at Nantong First People’s Hospital between January 2025 and June 2025 were selected as study participants. They were randomly assigned to either an experimental group or a control group, with 25 individuals allocated to each group using a random number table method.

Inclusion criteria were as follows: ① Completion of medical caregiver training at the time of employment and possession of a valid medical caregiver qualification certificate; ② Engagement in caregiving work for ≥ 6 months without having received retraining within the preceding 6 months; ③ Demonstration of effective communication skills, referring to the ability to communicate and interact normally with the instructors during the study; ④ Ability to operate a smartphone and use the WeChat application, the most widely used instant messaging application in China; ⑤ Age between 18 and 65 years; ⑥ Provision of informed, voluntary consent to participate in the study.

Exclusion criteria were: ① Absence from work due to sick leave or maternity leave; ② Provision of caregiving services who require more specialized professional training, such as those for pregnant women or infants. ③Withdrawal or resignation during the training period. Ethical approval for this study was obtained from the Ethics Committee of Nantong First People’s Hospital (Ethical Approval No. 2023KT137).

### 2.3 Methods

#### 2.3.1 Establishment of a medical caregiver training team

A three-tier structure comprising of “team leader–instructors–lecturers” was established to organize the medical caregiver training team. The team leader, a chief superintendent nurse with over 20 years of nursing experience, served as the deputy director responsible for nursing education and training in hospital management. Three nurses qualified in standardized training for medical caregivers were designated as instructors, responsible for monitoring course implementation and providing feedback to the lecturers. Eight contract lecturers with extensive teaching experience were assigned to serve as lecturers responsible for direct delivery of the training. The program covered four major modules, including professional cognition and regulations, basic knowledge of geriatric care, daily care and emergency skills, and safety and infection control. Two lecturers were assigned to each module to ensure continuity of instruction while avoiding excessive workload on individual lecturers.

Training sessions on the application of the KWL chart and the Teach-Back teaching method were organized by the team leader for all team members. Instructors and lecturers were required to acquire a comprehensive understanding of the core concepts, implementation principles, procedural steps, and critical elements associated with both the KWL strategy and the Teach-Back method. Both groups received instruction using a “one lesson, two lecturers” format to ensure consistency in teaching content and delivery. All lecturers had extensive teaching experience and were required to complete a trial teaching session prior to formal instruction to ensure teaching quality. A training session on the “KWL strategy and Teach-Back method” was organized and led by the team leader, including theoretical instruction and role-playing exercises. Lecturers were required to pass an assessment conducted by the team leader before delivering the course. During the teaching process, the team leader and instructors monitored the quality of information extraction using KWL worksheets and conducted random inspections of classroom implementation to ensure adherence to the standardized teaching protocol.

One month prior to the commencement of the training program, collective lesson preparation sessions were arranged by the team leader. During these sessions, instructors and lecturers collaborated to develop training courseware and establish a consensus regarding the objectives and content of the training curriculum. Ongoing monitoring and guidance of the training implementation process were overseen by the team leader.

The team leader continuously performed monitoring and guidance, organizing a meeting once per week. The three instructors reviewed the completed KWL charts weekly to ensure they were filled out correctly. After each session, they held a summary discussion with the lecturers to address any issues that arose and provided corrective feedback when deviations from the protocol were identified.

#### 2.3.2 Training protocol

Training approach: Given that medical caregivers were unable to leave their posts entirely for full-time training, and that most had relatively limited formal education, a hybrid training model incorporating both online and offline components was implemented. ① Online training: Online instruction was conducted jointly for participants in both the experimental and control groups. Instructional materials were provided in video format, with content delivered in clear, accessible language. A WeChat group was established for the dissemination of video QR codes, enabling repeated viewing of instructional materials, peer discussion, and open communication among participants. Scheduled online guidance and responses to trainee inquiries were provided by lecturers. ② Offline training: Offline sessions were conducted separately for the experimental and control groups in different classrooms. Each thematic session was delivered twice by the same lecturer to ensure consistency. These sessions focused on reviewing and reinforcing key content introduced during the online modules. The course consists of four modules: “Professional Awareness and Regulations,” “Basic Knowledge of Geriatric Nursing,” “Daily Living Care and First Aid Skills,” and “Safety and Hygiene Protection,” totaling 36 class hours. Among them, Professional Awareness and Regulations accounts for 3 class hours, Basic Knowledge of Geriatric Nursing for 10 class hours, Daily Living Care and First Aid Skills for 20 class hours, and Safety and Hygiene Protection for 3 class hours. The retraining program lasted 12 weeks. The language of the intervention was Chinese.

Teaching method for the control group: A traditional lecture-based model was used for the control group. Instructors provided theoretical instruction and operational demonstrations in accordance with the retraining syllabus, while medical caregivers engaged in passive knowledge acquisition. Theoretical instruction was held on Monday afternoons and operational demonstrations on Thursday afternoons. The sessions were scheduled in the afternoon because ward-related workload is generally lighter than in the morning, which helped minimize disruption to routine clinical work.

Teaching method for the experimental group: ① K stage (Activating prior knowledge) and W stage (Clarifying learning objectives). One week prior to each training session, KWL charts were distributed to participants, who were instructed to complete the “K” (Know) and “W” (Want to know) sections anonymously. Completed charts were collected within 3 days. The submitted responses were reviewed by instructors and lecturers to assess prior knowledge and identify gaps relevant to the upcoming topic. Instructional content was then adjusted accordingly to address identified deficiencies. ② L stage (Teach-Back for reinforced learning). Step-by-step instruction: After the delivery of key theoretical content, participants were asked to restate the material using their own words. For instance, prompts such as “Please explain the correct depth and frequency of chest compressions during cardiopulmonary resuscitation” were used to assess understanding. Immediate feedback: In cases of incorrect or incomplete responses, immediate correction and re-explanation were provided by the lecturer until accurate comprehension was demonstrated. Skill practice: A “demonstration–imitation–feedback” model was used during operational training. Following the execution of a procedure, caregivers were required to verbally describe each critical step. Performance was assessed by lecturers, and targeted guidance was offered as needed. Summary and reinforcement: At each session’s conclusion, participants completed the “L” (Learned) section of the KWL chart and evaluated whether previously stated questions had been resolved and learning goals were achieved. Subsequently, instructors and lecturers summarized the effectiveness of the session. Loose-leaf teaching materials were used throughout the training process. Common issues identified during instruction were compiled and addressed through targeted reinforced teaching. Theoretical instruction was held on Tuesday afternoons and operational demonstrations on Friday afternoons.

### 2.4 Evaluation indicators

#### 2.4.1 Theoretical assessment

Theoretical examination was developed by the medical caregiver training team based on the content covered during the four training modules. An initial pool of 80 questions was drafted, and a pilot test was conducted with five medical caregivers who did not participate in the formal study. Based on the pilot test results and participant feedback, 60 questions were ultimately retained for the final assessment. In addition, three nursing education experts were invited to review the theoretical examination items for content appropriateness. The medical caregivers involved in the pilot testing were also asked to evaluate whether the wording of the questions was clear and free of ambiguity, after which minor wording revisions were made accordingly. The assessment tool was developed in Chinese. The topics in the examination included medical ethics and law, daily living care, care for common diseases, psychological care, rehabilitation guidance, trauma and first aid, and safety-related knowledge. A closed-book test with a total possible score of 100 points was administered twice: once prior to training (pre-feedback test) and once following the completion of training (post-feedback test).

#### 2.4.2 Skill assessment

The operational skill assessment form was adapted and simplified by the training team from the hospital’s original standardized evaluation criteria, with the scoring items consolidated into no more than 10 key procedural steps. During the pilot-testing phase, five medical caregivers who did not participate in the formal study were recruited to perform the procedures, and two external examiners independently evaluated their performance using the tool to assess inter-rater consistency. Ambiguous items were subsequently revised based on examiner feedback. The assessment tool was administered in Chinese.

The assessment form adopted a 100-point scale. Examiners who had received standardized training conducted on-site assessments. After the training completion, one skill was randomly selected for evaluation using a random number table method. The skills included, but were not limited to, oral care, bed shampooing, bed bathing, position changing, and single-person manual cardiopulmonary resuscitation. Randomization was performed independently by a secretary of the training team who was not involved in the teaching or assessment of that session, ensuring that neither the lecturers nor the participants knew which skill would be tested in advance. The evaluation was conducted on-site by trained examiners, and each evaluation was limited to a duration of 15 min. The average completion time for these procedures was approximately 8–12 min. Therefore, the 15-min time limit was considered sufficient to accommodate normal task performance for the vast majority of participants while also allowing adequate time for examiner scoring, thereby minimizing the risk of inaccurate assessment due to time pressure.

#### 2.4.3 Medical caregiver competence

Medical caregiver competence was assessed using the Self-Assessment Questionnaire on Medical Caregiver Competence developed by Li and Shi (32). This instrument evaluates five dimensions: theoretical knowledge, operational skills, social competence, professional attitude, and personal traits. The questionnaire consisted of 56 items, rated on a 5-point Likert scale (1 = not competent to 5 = fully competent), resulting in total scores ranging from 56 to 280 points. The instrument demonstrated a content validity index of 0.96 and a Cronbach’s *α* coefficient of 0.982. Data were collected from participants prior to the initiation of training and again within 1 week following its completion. This instrument was administered in Chinese.

### 2.5 Quality control

In consideration of the specific characteristics of the medical caregiver population and based on findings from preliminary site investigations, all members of the training team (including 3 instructors and 8 lecturers) underwent standardized instruction prior to the initiation of the study, covering the KWL strategy, Teach-Back method, and use of loose-leaf teaching materials, followed by a competency assessment. To improve participant compliance, detailed training schedules were disseminated in advance via a designated WeChat group. Attendance was recorded for each training session.

Upon completion of the program, participants who successfully passed the assessments were issued qualification certificates and granted corresponding incentives (certificates were awarded to the top five performing medical caregivers in each group, five in the control group and five in the intervention group, and each recipient received a monetary reward of 100 RMB). During examinations, caregivers who had trouble with reading or written expression were supported by proctors who read the questions aloud and recorded the responses as dictated. After each test, answer sheets were reviewed immediately to verify that all items had been completed prior to submission.

### 2.6 Statistical methods

Data were processed using SPSS 25.0 statistical software. Descriptive statistics were used to summarize demographic variables. Categorical data were expressed as frequencies and percentages and analyzed using the chi-squared (χ^2^) test. Continuous variables meeting the assumption of normal distribution were presented as mean ± standard deviation (M ± SD) and analyzed using independent-samples *t*-tests or paired-samples *t*-tests, as appropriate. A value of *p* < 0.05 was considered statistically significant. Only one participant in the experimental group withdrew during the study period and was therefore excluded from the analysis. Complete case analysis was therefore used, and all reported analyses are based on the 49 completers (experimental group: *n* = 24; control group: *n* = 25).

## 3 Results

### 3.1 General data

A total of 49 medical caregivers were enrolled in the study. No statistically significant differences were observed between the experimental and control groups in terms of general demographic characteristics (*p* > 0.05). Detailed demographic data are presented in Table 1.

**Table 1:** Comparison of general characteristics (*n* = 49).

Items	Characteristics	Experimental group (*n* = 24)	Control group (*n* = 25)	Total (*n* = 49)	t/χ^2^ value	*p*
Age (years)		52.85 ± 5.00	52.79 ± 3.83	52.82 ± 4.40	−0.045	0.965
Sex	Male	9(37.50%)	6(24%)	15(30.61%)	1.051^b^	0.305
	Female	15(62.50%)	19(76%)	34(69.39%)		
Marital status	Unmarried	0(0%)	0(0%)	0(0%)	-^a^	0.609
	Married	22(91.67%)	24(96%)	46(93.88%)		
	Others	2(8.33%)	1(4%)	3(6.12%)		
Years of work experience		5.42 ± 2.89	5.44 ± 2.95	5.43 ± 2.89	0.028	0.978
Education	Primary school or below	10(41.67%)	9(36%)	19(38.77%)	1.260^b^	0.533
	Junior high/secondary technical school	9(37.5%)	13(52%)	22(44.90%)		
	High school/college	5(20.83%)	3(12%)	8(16.33%)		
	University or above	0(0%)	0(0%)	0(0%)		
Residence	Urban	18(75%)	16(64%)	34(69.39%)	0.698^b^	0.404
	Rural	6(25%)	9(36%)	15(30.61%)		

^a^Fisher’s exact test; ^b^χ^2^ test.

### 3.2 Comparison of theoretical knowledge prior to and following training

At baseline, theoretical assessments were administered to both groups. The mean theoretical knowledge scores were 71.08 ± 3.64 in the experimental group and 70.48 ± 2.73 in the control group, with no statistically significant differences observed (*p* > 0.05). One participant in the experimental group withdrew during the study period, resulting in a total of 49 participants completing the study.

Following 3 months of retraining, a post-intervention theoretical assessment was conducted. Improved scores were observed in both groups; however, the experimental group demonstrated significantly higher theoretical knowledge scores compared to the control group (*p* < 0.05). Detailed results are presented in Table 2.

**Table 2:** Comparison of theoretical knowledge and operational skills before and after training (*M ± SD*, points).

Group	Cases (n)	Theoretical knowledge- Prior	Theoretical knowledge -Following	*t*	*p*	Operational skills-Prior	Operational skills -Following	*t*	*p*
Experimental group	24	71.08 ± 3.64	84.04 ± 3.78	−20.670	<0.01	75.08 ± 5.45	88.42 ± 3.68	−13.415	<0.01
Control group	25	70.48 ± 2.73	76.08 ± 4.11	−8.257	<0.01	75.64 ± 5.78	84.40 ± 3.97	−8.374	<0.01
*t*		−0.659	−7.046			0.346	−3.669		
*P*		0.513	<0.01			0.730	<0.01		

### 3.3 Comparison of operational skills prior to and following training

Prior to the intervention, operational skill scores were 75.08 ± 5.45 in the experimental group and 75.64 ± 5.78 in the control group, with no statistically significant differences between groups (*p* > 0.05). After completion of retraining, operational skills were reassessed. Both groups exhibited significant improvement in performance; however, the experimental group achieved higher scores than the control group, with the difference reaching statistical significance (*p* < 0.05). Detailed results are presented in Table 2.

### 3.4 Comparison of medical caregiver competence prior to and following training

Baseline self-assessment scores for medical caregiver competence were 148.42 ± 5.02 for the experimental group and 148.36 ± 5.03 for the control group, with no statistically significant differences observed (*p* > 0.05). Post-training self-assessment indicated improvements in overall competence scores in both groups, with the experimental group scoring significantly higher than the control group (*p* < 0.05).

Further analysis of the five competence dimensions showed that post-training scores for theoretical knowledge, operational skills, social competence, and professional attitude were significantly higher in the experimental group compared to the control group (*p* < 0.05). No significant difference was identified between the two groups in the personal traits dimension. Detailed results are presented in Table 3.

**Table 3:** Comparison of medical caregiver competence before and after training (*M ± SD*, points).

Group	Number of cases (n)	Theoretical knowledge		Operational skills		Social competence		Professional attitude		Personal traits		Total score	
		Prior	Following	Prior	Following	Prior	Following	Prior	Following	Prior	Following	Prior	Following
Experimental group	24	23.13± 1.80	26.83± 1.71	52.25± 1.65	57.50± 2.11	21.25± 1.65	25.13± 1.42	37.54± 1.38	41.58± 1.47	14.25± 1.78	15.56± 1.71	148.42 ± 5.02	166.58± 5.73
Control group	25	23.24± 1.81	25.52± 1.96	52.28± 1.67	54.80± 2.04	21.28± 1.60	23.48± 1.76	37.44± 1.33	39.72± 1.51	14.12± 1.56	15.80± 1.55	148.36 ± 5.03	159.32± 5.96
*t*		0.223	−2.494	0.063	−4.557	0.065	−3.590	−0.263	−4.365	−0.272	0.548	−0.039	−4.346
*P*		0.825	0.016	0.950	<0.01	0.949	0.001	0.794	<0.01	0.787	0.586	0.969	<0.01

For the experimental group, paired comparisons prior to and following training across each dimension yielded the following *t*-values: −16.191, −14.903, −16.451, −14.837, −6.629, and −16.026, all *p* < 0.01. For the control group, *t*-values were: −16.808, −11.225, −17.041, −16.808, −13.394, and −19.530, all *p* < 0.01.

## 4 Discussion

This randomized controlled trial evaluated a retraining program for medical caregivers that integrated the Teach-Back method, the KWL strategy, and loose-leaf teaching materials, compared with conventional lecture-based instruction. Our results showed that the experimental group showed significantly higher post-training theoretical knowledge scores and practical skill scores. In addition, self-perceived competence improved in both groups, with significantly greater gains in the experimental group across four of five dimensions (theoretical knowledge, operational skills, social competence, and professional attitude, but not personal traits). These preliminary findings demonstrate that, in the short term, the integrated retraining model is more effective than conventional methods in improving knowledge, skills, and perceived competence among medical caregivers.

### 4.1 Effect of the Teach-Back method based on the KWL strategy and loose-leaf teaching materials on knowledge retention

Baseline assessments indicated that both theoretical and operational scores in the two groups were below 80 points prior to retraining. Despite prior completion of systematic training required for obtaining the “Medical Caregiver Qualification Certificate” and regular engagement in caregiving activities, there was clear evidence of knowledge attrition. Following retraining, improvements were noted in both theoretical and operational performance across groups, indicating that regular retraining contributes to enhanced knowledge retention (33).

Post-retraining assessment scores were significantly higher in the experimental group compared to the control group in both theoretical and operational domains (*p* < 0.05). This outcome may be attributed to the combined application of three instructional components: ① Loose-leaf teaching materials facilitated the connection of instructional tasks and allowed for flexible adjustments tailored to learners’ needs. ② The KWL strategy supported the activation of prior knowledge and promoted the integration of newly acquired information, enabling the establishment of associations between existing and novel content while reducing cognitive load. The strategy encouraged active inquiry, transforming the learning process from passive reception to active exploration. The processes of content summarization, reflection, and output further contributed to the structured reconstruction of knowledge (28). ③ The Teach-Back method incorporated repetition and immediate feedback to promote the recall and restatement of core concepts. This allowed for the identification of misunderstood or inadequately retained information, thereby reinforcing comprehension. These findings align with results reported in previous studies, supporting the practical applicability of the Teach-Back method when integrated with the KWL strategy and supported by loose-leaf teaching materials in the context of medical caregiver training (34).

### 4.2 Effect of the Teach-Back method based on the KWL strategy and loose-leaf teaching materials on competence development

The results indicated that self-assessed competence improved to varying degrees in both the experimental and control groups following the training, confirming the foundational role of systematic training in capacity building within the medical caregiving workforce (35). Notably, the experimental group exhibited a significantly greater increase in competence scores compared to the control group (*p* < 0.05). This enhancement was particularly evident in the domains of theoretical knowledge, operational skills, social competence, and professional attitude, indicating a qualitative distinction between conventional training approaches and the integrated retraining model incorporating the Teach-Back method based on the KWL strategy supported by loose-leaf teaching materials.

This integrated training model supports the development of a learner-centered, active, and closed-loop instructional process. Loose-leaf teaching materials contributed flexibility and personalized support; the KWL strategy established a structured cycle of “cognitive activation–motivation stimulation–reflective integration”; and the Teach-Back method facilitated the practical application and internalization of acquired knowledge and skills.

Among the five measured dimensions of medical caregiver competence, the personal traits domain was the only area where no statistically significant difference was observed between the two groups following the intervention. This outcome may be explained by the relative stability of personal traits, which are generally resistant to change over short-term educational interventions (36).

Several limitations in this study were identified. First, the study focused solely on immediate outcomes following retraining and did not include long-term follow-up assessments related to knowledge retention, skill durability, or patient care outcomes. To assess the sustained effectiveness of this approach, future research should incorporate longitudinal study designs. Furthermore, all participants were recruited from a single medical institution, and its specific training framework and management practices may have introduced institutional bias. To enhance the generalizability of findings, subsequent studies should consider larger sample sizes and include caregivers from multiple hospitals of varying levels and geographic regions. Thirdly, the control group received only conventional lecture-based instruction. While this provided a meaningful comparison against current practice, the experimental intervention combined three novel components (Teach-Back, KWL strategy, and loose-leaf materials). Therefore, we cannot determine the unique contribution of each component to the observed effects. A future factorial design or component-dissection study would be needed to isolate the effects of Teach-Back, KWL, and loose-leaf materials individually.

## 5 Conclusion

In conclusion, a retraining program that combines the Teach-Back method and the KWL strategy with loose-leaf teaching materials may effectively enhance learning outcomes, practical skill performance, and self-perceived competence among medical caregivers. This approach has potential for wider application in the clinical training of medical caregivers and may also provide useful insights for continuing education in nursing and other healthcare-related disciplines.

## Data Availability

The original contributions presented in the study are included in the article/supplementary material, further inquiries can be directed to the corresponding author.
